# Dr. Decatur P. Gregg

**Published:** 1867-11

**Authors:** 


*Obituary.*—As we go to press we are pained to learn of the death of Dr. Decatur P. Gregg of Columbia, S. C. Dr. Gregg was a graduate of the class of 1853, and was for a long time in successful practice at Charlotte, N. C. Of late years Dr. Gregg has been a resident of Columbia, S. C., where he was much respected for his high moral as well as professional worth. Dr. Gregg had been appointed one of the "Harris Lecturers" for the present session of the Baltimore College of Dental Surgery.

